# Maximizing respondent-driven sampling field procedures in the recruitment of sexual minorities for health research

**DOI:** 10.1177/2050312119829983

**Published:** 2019-02-17

**Authors:** Amy L Hequembourg, Christina Panagakis

**Affiliations:** 1 School of Nursing, University at Buffalo, Buffalo, NY, USA; 2 United Way of Buffalo and Erie County, Buffalo, NY, USA

**Keywords:** Respondent-driven sampling, sexual minorities, recruitment methods, hidden populations, health disparities research

## Abstract

**Objectives:**

Research to address the significant health burden experienced by sexual minority populations remains hampered by a lack of appropriate sampling methods to support evidence-based studies. Respondent-driven sampling offers one viable strategy to recruit these hidden populations. Because few studies systematically report their experiences using respondent-driven sampling to recruit sexual minorities, this article aligns with recent recommendations for the standardization of reporting and transparency in studies utilizing respondent-driven sampling. We (1) provide detailed descriptions about the successful execution of respondent-driven sampling in two community-based studies of sexual minority individuals, (2) outline procedures to enhance the effectiveness of respondent-driven sampling referral processes, (3) present mixed-methods results regarding the effectiveness of respondent-driven sampling in our studies, and (4) offer recommendations for other researchers when using respondent-driven sampling.

**Methods:**

We successfully recruited 655 sexual minority men and women for two studies using respondent-driven sampling.

**Results:**

Resulting metrics indicate the successful achievement of equilibrium in each study. In addition, exit interviews elucidated strategies to effectively target referrals who meet the study criteria and procedures to promote the study that will maximize referral chains and ensure attainment of equilibrium.

**Conclusion:**

Mixed-methods results suggest that respondent-driven sampling can be an effective means of recruiting a community-based sample of sexual minorities in smaller urban regions. Limitations are presented and suggestions are offered to researchers utilizing respondent-driven sampling in future studies.

## Introduction

Since its inception, respondent-driven sampling (RDS) has become a popular tool for recruiting a variety of hidden populations, including sexual minorities.^1,2^ Prominent funding agencies, including the National Institutes of Health (NIH), Centers for Disease Control and Prevention (CDC), and World Health Organization (WHO), have supported studies utilizing RDS. Despite its popularity, there are a series of reporting gaps that hamper field advancement, prompting efforts to standardize reporting across studies.^3,4^ First, few concerted efforts have been made to offer transparency in the RDS recruitment process. Thus, many studies fail to provide important details about the implementation and objective utility of RDS.^5^ In addition, less is known about the effectiveness of RDS in the recruitment of sexual minorities residing in smaller cities lacking clearly delineated sexual and gender minority communities. The bulk of RDS research has occurred in large metropolitan or international settings with densely networked target populations, often on the topic of HIV surveillance.^4,6–11^ In addition, far fewer researchers have utilized RDS to recruit women, and—with one exception^12^—no known studies have specifically addressed the use of RDS in the recruitment of health-related research with sexual minority women. These limitations hamper the ongoing investigation of health disparities among sexual minorities.

More than 9 million adults in the United States identify as lesbian, gay, bisexual, or transgender (i.e. sexual minorities^13^), and they suffer a disproportionate health burden compared to exclusively heterosexual individuals. In response to these persistent disparities, the Director of the National Institute of Health’s Institute on Minority Health and Health Disparities designated gender and sexual minorities as a health disparities population in October 2016. This designation represented a critical public health juncture whereby research aimed at understanding health inequities among gender and sexual minorities are targeted for promotion and fiscal support in coordinated efforts similar to those that are applied to other vulnerable populations. However, an ongoing challenge associated with studying this population is the lack of visible markers designating sexual minority status from the majority. The “hidden” nature of sexual minority populations is a significant challenge for recruiting them to participate in research studies. Meyer and Wilson^14^ point out that, “Sampling of study participants has probably been one of the most important methodological factors influencing the evolution of research on lesbians, gay men, and bisexual men and women” (p. 23). As sexual minorities are an important population for study, but often difficult to find and recruit, we respond to calls for transparency to improve future RDS studies by providing a detailed overview of our effective use of RDS to recruit sexual minorities in a small urban city in the Northeastern United States. We also present results regarding the effectiveness of RDS in our studies, including qualitative insights about participants’ experiences using coupon referrals.

### What is RDS?

RDS is a particularly attractive recruitment method for sampling sexual minorities for research purposes because it utilizes a participant-driven referral incentive system to decrease sampling bias in the recruitment of hidden populations.^1^ RDS begins with initial “seed” participants who form the first wave of the sample. There are no formally prescribed methods for selecting the specific seed participants, and the characteristics of the seed participants should, theoretically, be irrelevant if equilibrium is achieved.^1,15^ Equilibrium—the ultimate metric of a successfully executed RDS study—refers to the maximization of referral waves to the point where the sample composition stabilizes and becomes independent of the seeds.^16^ However, some researchers advocate for the strategic selection of seeds to facilitate the initiation of productive and diverse referrals chains.^17,18^

Once a seed participant instigates a referral, each subsequent participant is provided with nominal monetary incentives to refer friends to the study. This method of peer referrals eliminates the ethical dilemma associated with asking respondents to divulge sensitive information about their peers to researchers who would then directly contact the referrals.^1,19^ Participants are issued unique referral coupons and those coupons are used to trace recruitment patterns in the population. The goal is to use a minimum number of seed participants while maximizing the number of referral waves in order to result in a sample composition that is independent of the initial seed respondents.^1^

Several functional and analytic assumptions are required for the effective execution of RDS.^18,20^ The first functional assumption is that, as members of the target population, respondents have to be networked sufficiently in order to be able to refer one another. Second, networks within which participants are embedded must be sufficiently large and densely networked to facilitate ongoing recruitment. Third, sampling with replacement must be theoretically possible, so that each participant could potentially be recruited multiple times by different individuals within the network. In practice, however, study participants are only allowed to participate once so as to sustain multiple waves of recruitment. In addition to these functional requirements, RDS also requires the fulfillment of several analytic assumptions. Respondents must be able to (1) accurately estimate and report on their network size and composition, (2) recruit randomly from their networks, and (3) recruit at least one peer from their networks (also see Lee et al.^21^ for additional RDS assumptions to consider). Fulfillment of these functional and analytic assumptions results in an unbiased sample in which resulting population estimates are asymptomatically unbiased. Careful planning is warranted in the design and use of RDS for recruitment purposes since violations of these functional and analytic assumptions will compromise the effectiveness of RDS in unanticipated ways.^14,21,22^ In the following pages, we outline our use of RDS in two studies of sexual minority participants, providing a detailed account of the procedural elements that ensured our successful use of RDS.

## Methods

### Study 1: Project COPE

Our first study (i.e. Study 1: Conversations on Personal Experiences, or Project COPE), funded by the NIH (K01 AA016105 [PI Hequembourg]), was a cross-sectional study to examine correlates associated with alcohol use and experiences of interpersonal violence using surveys and qualitative interviews. Participants included nearly 400 sexual minority men and women (approximately 100 each gay men, bisexual men, lesbian women, and bisexual women). Twenty seeds (five each of gay men, lesbians, bisexual men, and bisexual women) were initially recruited using different approaches, including advertisements in a local entertainment newspaper, recruitment flyers, and by word-of-mouth. Recruitment flyers sought individuals who self-identified as lesbian, gay, or bisexual and were between the ages of 18 and 35 years old who would be interested in sharing their stories “about everyday hassles you experience and the ways you manage them.” Transgender men and women were ineligible. Following a telephone screening to determine eligibility, participants were scheduled to visit the Research Institute. Participants who called the study as a referral were asked to provide the unique serial number from their referral coupon and their relationship to the referrer. Scheduling and reminders were mailed via the US postal service.

Participants completed an extensive in-person baseline self-administered survey and interviewer-administered timeline followback assessments about recent substance use and qualitative interviews about experiences of interpersonal violence and experiences of sexual assault (when applicable). Results from these studies, pertaining to lifetime victimization experiences of sexual minorities recruited using RDS, are presented elsewhere.^23,24^ Informed consent, which was approved by the University at Buffalo Institutional Review Board, was obtained from all individual participants included in the study.

**Figure 1.**
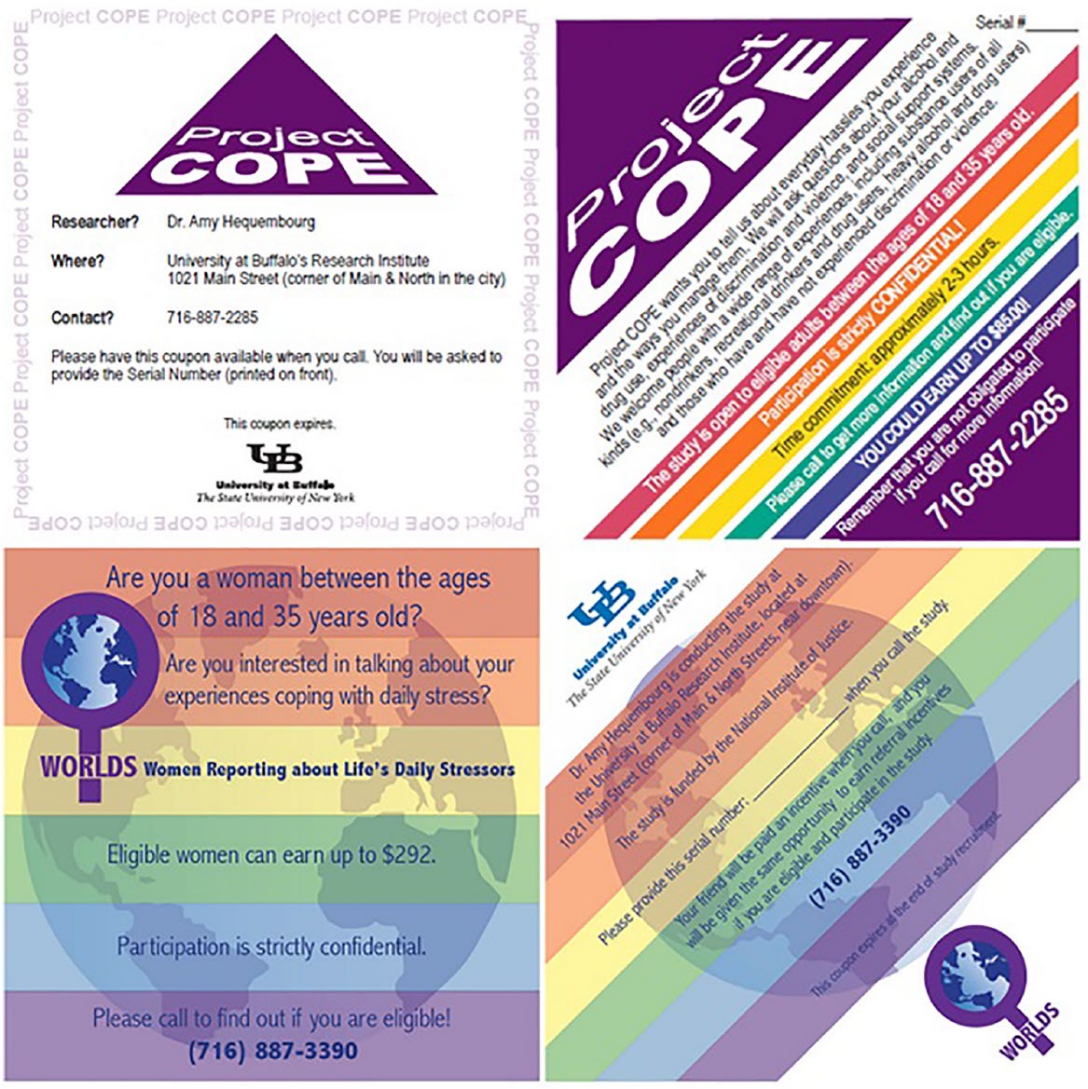
Referral coupons.

In-person assessments lasted 2–3 hours and participants were compensated US$50. Upon completion of the assessment, participants were provided with three hard-copy referral coupons (Figure 1) and invited to distribute them to three of their gay, lesbian, or bisexual friends who lived in the local region and were between the ages of 18 and 35. They were instructed to only refer friends rather than new acquaintances or strangers. We also cautioned participants that—because the study only included gay, lesbian, and bisexual individuals—they would be disclosing their own sexual minority status to anyone who they referred. Based on nominal referral fee structures utilized in other RDS studies,^17,25,26^ participants were offered US$10 for each of three referral coupons plus an additional US$5 “steering incentive”^15^ if they referred one self-identified bisexual man or woman. Although participants were told to use their coupons to recruit their gay, lesbian, or bisexual friends to the study, the actual referral coupon did not reference sexual identity to avoid unintentional disclosure of the participant’s sexual identity to someone who saw the coupon in his or her possession.

Each participant was asked to report his or her network size. We asked each participant to report the numbers of (1) gay men, lesbians, and bisexual men and women; (2) identity-specific individuals (e.g. gay men asked to report number of gay men); and (3) heterosexual individuals in their social networks. Population parameters reported in this article were computed using the network responses for the combined gay men, lesbians, and bisexual men and women.

### Study 2: WORLDS

Project COPE informed a subsequent grant funded by the National Institute of Justice (2014-VA-CX-0067 [PI Hequembourg]) to identify unique mechanisms associated with sexual assault among sexual minority women compared to heterosexual women. In this study (i.e. Study 2: WOmen Responding to Life’s Daily Stressors or WORLDS Study), we recruited nearly equal numbers of heterosexual, bisexual, and lesbian women using RDS to participate in a longitudinal study involving baseline surveys, daily diary reports, and qualitative interviews. A total of 15 seeds (5 each of lesbian, bisexual, and heterosexual women) were initially recruited for this study via flyers posted in LGBTQ-related community locations, including a LGBTQ community center and university wellness services listserv. Recruitment materials asked women who were “interested in talking about daily stress” to contact the WORLDS Study by phone to determine eligibility. Callers were also asked to describe their relationship to the person who referred them. After participants were determined to be eligible, they were scheduled to complete baseline surveys in-person at our Institute. Informed consent, which was approved by the University at Buffalo Institutional Review Board, was obtained from all individual participants included in the study.

During this visit, participants received a detailed explanation of the referral process. Participants also were encouraged to share their coupon referrals using electronic means, such as sharing photos of their coupons via text. The WORLDS Study FAQ sheet provided participants with sample text that they could use to refer friends via social media, including Facebook and Twitter. They were instructed to ask their friends to contact them directly to seek the referral coupon code that they would then report to study staff to facilitate the incentive payment. They also were each given four printed, paper copies of the referral coupon (see Figure 1), but offered incentives for a maximum of *three* friend referrals. This buffer allowed participants to attain their referral maximum even if one peer decided not to participate. Each participant could earn US$10 per referral, up to a maximum of US$30. If a referral identified as a woman-of-color, the referrer was given a one-time US$5 steering incentive. While allowing participants to distribute four coupons did create the possibility that a participant could refer more than three friends to the study, only a small minority of women (n = 9) did so. In each of those cases, the women were only paid for their first three referrals. Following standard RDS protocols, participants were asked to report their network size, including the size of their (1) lesbian and bisexual female social network and (2) the size of their identity-specific network (e.g. bisexual women reported about the size of their bisexual female social network). For the purposes of this article, we utilized women’s reports about their lesbian and bisexual networks to estimate the population parameters.

The intensive, longitudinal nature of this study ensured that staff were regularly in contact with participants for the duration of the data collection period (12 weeks). Participants were mailed a check bi-weekly and we included individualized letters in those mailings that detailed their earned incentives and provided a reminder about the number of unclaimed referral incentives available to them. In addition, every 8 days of the daily survey inquired if they needed additional referral coupons. Additional coupons were only distributed to participants who had not yet been incentivized for three referrals. We chose to allow participants to request more than the initial four coupons they received at baseline in recognition of the likelihood that some participants’ friends would take a coupon but never call the study to participate. Rather than requiring participants to pursue the same few peers who may not be interested in participating, we preferred to allow them to randomly choose other members of their peer network to refer.

As we approached our target quota in each of the sexual identity categories, we alerted participants that we would provide them with a referral incentive if their friends called the study, but their friends would not have the opportunity to participate if we had reached our target recruitment quota in that particular category. At the end of the 12 weeks, participants were approached by phone and email to identify those willing to participate in a brief interview about their referral experiences. Qualitative RDS interviews were conducted by the second author, in her capacity as Project Director on the WORLDS Study, and another staff member. Second author, Panagakis, holds a PhD in Sociology and has extensive experience conducting qualitative interviews, while the staff member has a clinical Master’s degree and received extensive training from both authors in the conduct of qualitative interviews. Phone interviews were audiorecorded and transcribed by study staff. Transcription accuracy was confirmed by the first author.

### Data analyses

Adjusted proportion estimates were computed using 95% confidence intervals and adjusted mean network sizes using RDS Analysis Tool (RDSAT) Version 7.1.46. Recruitment patterns reflected who recruited whom, which were tracked in each study using participant’s unique coupon numbers. Reported social network size (as described above for each study) was the metric for social network composition. These data were utilized to derive weights for computing proportion and variance estimates. Adjusted population proportions refer to the broader sexual minority in the small, urban city in the Northeastern United States in which we conducted these studies.

We assessed the effectiveness of RDS by computing proportion estimates, social network tie adequacy, transition probabilities (i.e. network homophily), and the attainment of equilibrium for our primary variable sexual identity. Adequate social ties were defined as mean network size ⩾3. Network homophily values range from −1 to +1, with lower scores (−1) representing exclusive recruitment of out-groups and higher scores (+1) representing exclusive in-group recruitment. Zero values indicate that social ties cross networks, suggesting that preferential group recruitment biases were overcome and contacts were randomly recruiting from the population of all available recruits.^1,2^ Equilibrium distributions were set at the RDSAT default (i.e. falling within 2% of the sample distribution).

Qualitative RDS interviews were audio-recorded, transcribed, and entered into Atlas.ti. Themes and classification systems were subsequently determined by the authors through a multistage inductive process.^27,28^ Open coding identified themes and categories, which were determined based on frequency, specificity, and extensiveness.^29^ Representative quotes were extracted to illustrate the themes.

## Results

### Study 1: Project COPE

**Figure 2.**
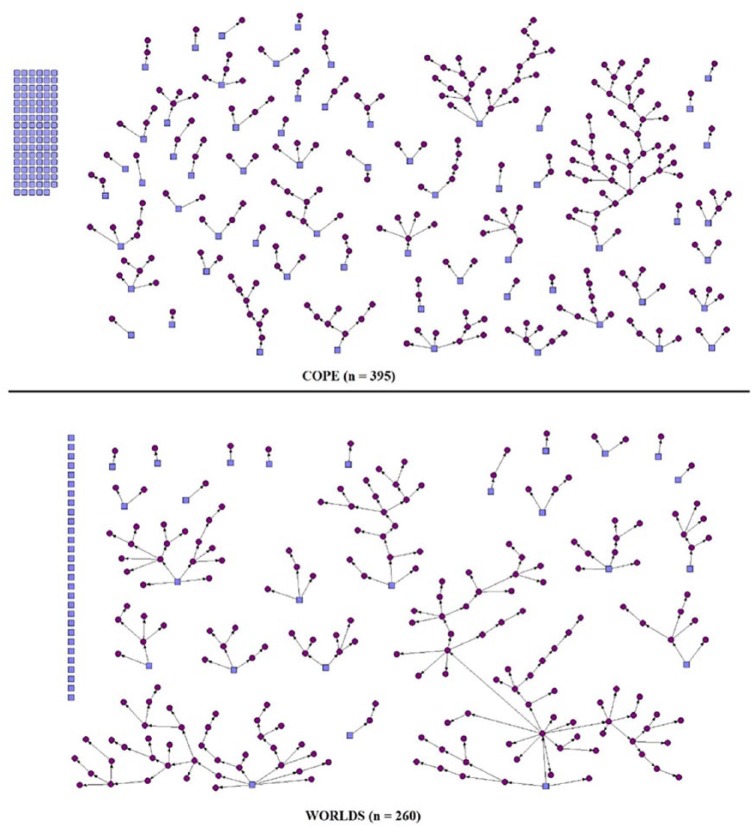
Referral network diagrams.

Between October 2007 and April 2010, 395 sexual minority men and women (103 gay men, 101 lesbian women, 86 bisexual men, and 105 bisexual women) were recruited using RDS to participate in Project COPE. Our final sample comprised 162 seed participants, which represented 41% of the total sample. As depicted in Figure 2, 68 of the seeds (or 42% of all seeds) referred someone to the study. A total of 1278 coupons were distributed to participants over the course of the study, 22% (n = 275) of which were redeemed.

Several lulls in referrals during the study required pursuit of additional seed participants using flyers and newspaper advertisements. We also direct-mailed active participants a brief survey in an attempt to motivate additional recruitment. The response rate was low (n = 17). When asked why they thought their friends had not volunteered for the study, the most common answers were that they didn’t know, that their friends kept telling them that they were going to call but then provided no reasons for not doing so, and/or their friends simply did not have time to participate. While 51 additional coupons were mailed to participants who requested them as a result of this mailing, only four new screening calls occurred as a result of this mailing.

Bisexual men were particularly difficult to recruit for the study, and they were the least likely to refer friends to the study. Many of them explained that they had disclosed their sexual identity in very small networks of other bisexual-identified friends and, therefore, did not feel comfortable distributing referral coupons to friends outside those networks. Consequently, the length of the recruitment period was greatly prolonged due to our ongoing efforts to recruit bisexual men. Ultimately, the final study sample included fewer bisexual men than we had originally planned.

Adjusted sample characteristics are presented in Table 1, including adjusted population proportions, mean network sizes, and homophily indices for each of the four subgroups. Equilibrium was reached by wave seven for sexual identity, the main variable of interest (Figure 2). The final sample comprised sexual minority men and women with large mean social network sizes (>20, Table 2), indicating numerous network ties to other sexual minorities based on sexual identity. Bisexual men reported the largest network size and bisexual women the smallest. Homophily indices (Table 1) indicated that there was low to moderate insularity, with lesbian and gay men most likely to refer from within their own sexual identity group, while bisexual men showed minimal preference for either in- or out-group referring. Referral patterns (Table 2) suggest that most occurred within similar gender and sexual identity groups, although there was some variability across groups. Gay men were most likely to recruit other gay men and least likely to refer lesbians, bisexual men were most likely to refer bisexual men and women and least likely to refer lesbian women, lesbian women were most likely to refer other lesbian women and least likely to refer bisexual men, and bisexual women were most likely to refer other bisexual women and least likely to refer gay men.

**Table 1.** Transition probabilities.

COPE (N = 395)				
Sexual Identity	Lesbian (n = 101)	Bisexual women (n = 105)	Gay (n = 103)	Bisexual men (n = 86)
Lesbian	0.68	0.23	0.07	0.03
Bisexual women	0.12	0.48	0.02	0.38
Gay	0.05	0.18	0.56	0.18
Bisexual men	0.09	0.42	0.12	0.42
WORLDS (N = 260)				
Sexual Identity	Lesbian (n = 88)	Bisexual women (n = 84)	Heterosexual women (n = 88)	
Lesbian	0.64	0.29	0.06	
Bisexual women	0.36	0.55	0.09	
Heterosexual women	0.14	0.19	0.68	

COPE: Conversations on Personal Experiences; WORLDS: WOmen Responding to Life’s Daily Stressors.

**Table 2.** Adjusted sample characteristics.

Sexual Identity	n	Adjusted population proportion (95% CI)	Mean network size	Homophily
COPE (N = 395)				
Lesbian	101	0.15 (0.07, 0.26)	40.05	0.62
Bisexual women	105	0.33 (0.22, 0.42)	21.26	0.22
Gay	103	0.12 (0.04, 0.21)	34.04	0.50
Bisexual men	86	0.39 (0.29, 0.56)	51.18	0.04
WORLDS (N = 260)				
Lesbian	88	0.32 (0.21, 0.47)	15.63	0.49
Bisexual women	84	0.31 (0.19, 0.41)	9.53	0.35
Heterosexual women	88	0.37 (0.20, 0.54)	4.92	0.49

COPE: Conversations on Personal Experiences; WORLDS: WOmen Responding to Life’s Daily Stressors; CI: confidence interval.

### Study 2: WORLDS Study

We recruited 88 lesbian, 84 bisexual, and 88 heterosexual women (N = 260) to participate in the WORLDS Study between October 2015 and May 2017. We began the study by recruiting 15 initial seed participants. New referrals dwindled a few times during the study (e.g. during the holidays) and prompted us to seek new seeds via advertisements and flyers. Twenty-one percent of our final sample (n = 54) comprised seed participants. Of those seeds, 26 were successful and 28 were unsuccessful in prompting referrals. Half (n = 127 or 49%) of women in the final WORLDS Study sample referred at least one friend to the study. In total, we distributed 1353 referral coupons, 20% of which (n = 277) were redeemed. Among the 260 participants, nine women referred more than three friends to the study. One woman referred ten friends, two women referred six friends, one woman referred five friends, and four women referred four friends. However, five of those eight referrers were in chains that did reach equilibrium, which suggests that while we did have a small percentage of our sample over-refer, it did not adversely impact our ability to reach equilibrium.

In Table 2, we provide adjusted sample characteristics including adjusted population proportions, mean network sizes, and homophile indices for each of the three subgroups. Equilibrium was reached by wave seven for the sexual identity variable (Figure 2). The final sample comprised sexual minority and heterosexual women with relatively small social networks (<16; Table 1), indicating low network ties to other women based on sexual identity. Heterosexual women reported the smallest social networks and lesbian women the largest. Referral patterns (Table 1) showed that women were most likely to refer friends of the same sexual identity. Lesbian and bisexual women were least likely to refer heterosexual women, while heterosexual women were least likely to refer lesbian women. Homophily indices indicated that there was moderate insularity, with participants—regardless of sexual identity—likely to refer from within their own sexual identity group.

At the end of their participation in the study, we contacted participants via phone or email to learn more about their experience with the RDS process. We were especially interested in understanding how the referral process worked. For example, we wished to shed light on subsequent steps taken by participants to refer friends after they received training from our staff on how the referral process worked. These interviews, which lasted 5–10 min each, included questions about their method for selecting friends to target for referrals, their strategies for promoting the study in their social networks, and the logistics through which they delivered referral coupons. Ultimately, we spoke with 163 participants, or 63% of the total sample who indicated interest and availability. Of those 163 women, 66 identified as heterosexual, 48 identified as lesbian, and 49 identified as bisexual. Representative quotes are presented in Table 3 and discussed in greater detail below.

**Table 3.** Qualitative responses.

Selection: how do participants choose friends to refer to the study?	
Study criteria (63 participants)	Friends in network (30 participants)
*I chose my friends who I knew were bisexual because I knew there were already a lot of straight women in the study.* (ID 11865, bisexual) *Well, I chose people based on who the study needed so I asked you what populations you were lacking and I tried to find people who I knew who met those needs.* (ID 10897, bisexual)	*It was pretty much just people I came into contact with in my everyday life.* (ID 10665, heterosexual) *Well actually, I ended up hanging out the group of friends that weekend and brought it up to them that I was in the study and they seemed interested so I gave them the coupons.* (ID 11185, lesbian)
Promotion: how do they describe the study to their friends?	
Study logistics (67 participants)	Incentives (54 participants)
*I told them about my experience. I went into the initial survey, they ask you questions about your demographics and about your previous history. Not the full survey questions, but just a general sense of the questions they would ask.* (ID 11937, lesbian) *I explained a little bit more in depth how long it took, how easy it was, that it was personal but you made me feel at ease, that it is a good study and that as women, we should worry about our daily interactions. We are so accustomed to being harassed that you don’t even notice it.* (ID 10217, heterosexual)	*I told her yes you’re going to make a little bit of money when you do the survey, but it’s not about that. It’s about filling out the survey and saying what you feel, whatever the case is. The money part was not the first thing I told her. But then when I told her the money she was like, okay.* (ID 10145, heterosexual) *I was like hey here’s what I did, it’s legit, it’s for UB. I sent them photos of some of the paperwork that you guys had provided me on my visit with kind of the payout scales a recap of what it was. And I just sent them all of that and then I sent them a picture of the check I got when I came in and I was like, no seriously I just went today and I got a $25 check.* (ID 10561, bisexual)
Logistics: how do they distribute the referral coupons?	
Hybrid of in-person and mediated communication styles (107 participants)	
*For most of the people I hand delivered. I think for if I wasn’t going to see them I actually just took a picture of it with my phone and texted it, but for most of them I saw them in person.* (ID 11153, heterosexual) *I actually just posted something on Facebook, using the language that you had given me. Then I think I got contacted by 50 people or so. […] I crafted a little message to use to reply back to them and just saved it on my desktop. And so then as people sent me individual messages or posted on my Facebook wall I had language about how then they were supposed to contact you guys.* (ID 10601, heterosexual)	

When discussing how they selected members of their networks to refer to the study, participants spoke about two themes. First, they considered which members of their network met our study criteria. Participants developed an understanding of our selection criteria for the study because we spent time during the in-person visit to explain our rationale, including the importance of recruiting three same-size samples of women by sexual identity. For example, one woman told us, “I chose my friends who I knew were bisexual because I knew there were already a lot of straight women in the study.” This suggests that they did not randomly select referrals, but instead took time to consider who would best meet our goals. The second theme reiterates that these women did not choose referrals randomly. When asking about their relationship to the women they chose to refer, the most common answer was that they chose friends rather than acquaintances. This theme is especially important because it demonstrates that our participants followed the assumptions underlying RDS theory, namely that referrals must occur within their personal social network. We also purposely offered nominal referral incentives so as to avoid encouraging quick and thoughtless referrals pursued by participants simply for financial gain, which could have resulted in referrals of strangers or individuals on the fringes of their networks. Instead, our qualitative data confirmed that the women in our study referred other women who were part of their social networks.

Participants described two common ways that they explained the study to their friends during the referral process. First, many women focused on the study logistics, including the study criteria, duration of study participation, and/or the different stages of data collection. They did this as a way to make sure their friends were prepared and knew what our study involved before calling us to see if they were eligible. Second, they highlighted the financial incentives participants received from taking part in our study. For example, a participant described telling a friend, “I just sent them … a picture of the check I got when I came in and I was like, no seriously I just went today and I got a $25 check.” While a few women talked about feeling it was important to participate in our study regardless of compensation, many more indicated that financial incentives were an important motivation for participation.

Finally, we asked about the referral logistics, in particular, the methods they used to distribute the referral coupons to their friends. While we provided four paper coupons to the participants, they explained that they often did not rely solely on these paper versions to distribute to their friends. Instead, they talked about using a hybrid of methods depending on the circumstances. While some women distributed coupons in person, this was not always the case. One participant described, “For most of the people I hand delivered. I think if I wasn’t going to see them I actually just took a picture of it with my phone and texted it.” Texting a photo to friends was a very common response, as many women indicated that they might not see their friends in person on a regular basis, but they were still in contact via texting. They also described using social media to refer friends, for similar reasons as texting. Of all social media platforms, Facebook was the most commonly used for referrals. Utilizing electronic methods opened up referral possibilities above and beyond what could be achieved in person. It allowed women to choose members of their network who they believed be a good fit for the study but would be difficult to reach if they could only distribute the coupon in person.

## Discussion

Recruiting sufficient numbers of participants from hidden populations for in-person assessments remains an overriding challenge for researchers seeking to either recruit in smaller population areas or better understand the mechanisms associated with health disparities among sexual minorities. Given the strengths of RDS as a recruitment strategy to effectively recruit hidden populations, this article offers a transparent look at our successful use of RDS to recruit sexual minorities in a smaller, urban city. Project COPE and the WORLDS Study resulted in a combined recruitment of 655 sexual minority men and women. RDS metrics, including the successful achievement of equilibrium in each study, suggest that RDS can be effectively used to recruit a community-based sample of sexual minorities in geographic regions of the United States that contain less clearly defined sexual minority communities than those found in larger metropolitan areas (e.g. New York City, San Francisco).

There were several key differences between our two studies that held consequences for the procedural aspects of RDS. First, the two study designs differed significantly, with Project COPE using a cross-sectional approach and the WORLDS Study using a prospective, longitudinal approach. An immediately discernible benefit of the longitudinal design of WORLDS was that it naturally facilitated ongoing interactions with participants that helped promote good will and sustained interest in referring friends to the study. A second difference between the two studies was that the WORLDS Study included significantly more staff coverage than Project COPE, thus allowing for more expedient and consistent response in the former study. The final significant difference was that Project COPE was conducted prior to the popularity of smartphone usage that was commonplace among WORLDS Study participants. Thus, we were able to encourage participants to utilize technology to share their referral coupons in the WORLDS Study, compared to participants’ reliance on paper coupons in Project COPE. Email also was much more commonly utilized by WORLDS Study participants compared to Project COPE participants and, thus, reminders and other prompts were more easily facilitated in the WORLDS Study than via the postal services methods used in Project COPE.

### In the participant’s own words

The WORLDS Study provided the opportunity to collect novel qualitative findings about participants’ experiences utilizing RDS. This study provides insights regarding RDS from the perspective of the participants. These data provide critical insights into the firsthand experience of participating in a referral-based study, confirming or disavowing the logistical RDS assumptions that researchers make regarding this process. Allowing respondents to share, in their own words, how they referred members of their social network to the study illuminates *how* the referral process actually occurs and is an important contribution to efforts in the literature to provide transparency in RDS reporting. Responses underscored the usefulness of our procedural efforts. For example, women confirmed that they were referring their friends rather than acquaintances or strangers, which is integral to the successful assessment of the population proportions based on their reports of network size. In addition, their answers indicated that the time we spent teaching them about the referral process, in tandem with versatile methods for distributing coupons, led to an ease when approaching friends about participation. These interview responses underscore the importance of dedicated staff who thoroughly explain the research participation requirements (e.g. reporting expectations, target sample characteristics to inform referrals) and convey a culture of enthusiasm and support for the referral process. We also learned during the course of our two studies that mass correspondences with participants to encourage coupon referrals and distribute additional coupons are largely ineffective. The longitudinal nature of the WORLDS Study suggested, however, that routine contact with participants encouraged greater efforts to distribute coupons, although those attempts were not always effective. In sum, participants were prepared to refer friends because study staff equipped them for the task.

### Contextualing results concerning seed participants

Based primarily on the greater number of seeds required for Project COPE versus the WORLDS Study (162 vs 54), we initially postulated that the latter represented a more effective use of RDS in the recruitment of sexual minorities from our local community. We reasoned that we had fewer seeds in the WORLDS Study due to improved staffing that afforded participants more expedient access to the screening process and interview scheduling than was available in Project COPE. We also surmised that the differing study designs (WORLDS: longitudinal vs COPE: cross-sectional) fostered ongoing interactions with participants over time in the WORLDS Study that facilitated the referral process in ways that were not possible in the cross-sectional COPE Study. However, after conducting our RDS analyses, we revised our initial interpretation. Improved staffing for WORLDS did not result in noteworthy differences from Project COPE in the length of the recruitment periods, our ability to reach equilibrium in each study, or in the number of successful seeds in the WORLDS Study and Project COPE. However, there is some evidence to suggest that it did help to create longer, more robust referral chains in WORLDS compared to Project COPE (Figure 2). It is also entirely possible that the longitudinal nature of the WORLDS Study and the repeated interactions with staff over the course of the study may have inadvertently introduced biases into the participant selection process, whereas the unfettered nature of the COPE Study recruitment process may have allowed referrals to emerge in a more organic fashion. Our revised conclusions based on the final RDSAT results underscore the importance of introspection on the part of researchers to understand how study protocols may influence the success or failure of their RDS recruitment efforts. Our final interpretation of the results is that each study utilized this recruitment approach successfully from the perspective of the RDS metrics (e.g. length of referral chains and attainment of equilibrium), but with some important distinctions in the processes and outcomes by which we attained those results. In the remainder of this discussion, we outline some possible limitations of our RDS strategies and propose potential solutions for future research using RDS to recruit sexual minority samples.

### Concerns and potential limitations

The state of knowledge regarding RDS is swiftly evolving, including the introduction of new techniques for strengthening and extending its utility (e.g. RDS web-based approaches).^30,31^ With the increasingly prolific use of RDS, researchers have voiced a variety of concerns regarding the basic assumptions underlying RDS.^21,22,32^ In consideration of that growing literature for the current discussion, we discuss a number of potential limitations in the following paragraphs and conclude with suggested readings to improve future studies using RDS for recruitment of hidden populations.

Our use of RDS for recruiting sexual minorities in a smaller urban area resulted in longer recruitment periods than found in the literature. A review of HIV surveillance studies (WHO, 2011) conducted using RDS found remarkably swift data collection periods ranging from 3 to 14 weeks to recruit, in some cases, up to 530 participants. In contrast, our studies experienced some periods of high response volume that were interspersed with months of low referral activity. These lulls often required intervention by study staff who either contacted current participants with reminders about referrals and associated incentives or took steps to secure more seeds via a variety of recruitment strategies. Furthermore, in studies requiring the recruitment of different subgroups within the same study, researchers may find that some of those groups are more challenging to recruit than others and, thus, protract the overall recruitment time (e.g. the recruitment of bisexual men in Project COPE). Although the rapidity of recruitment using RDS for HIV surveillance methods is highly attractive, our experiences suggest that researchers carefully consider their target population and recruitment venue and plan accordingly when estimating the time frame needed to reach their target sample size and equilibrium.

Our strategies for coupon distributions also may have had indeterminate consequences for the quality of our final referral networks. In the WORLDS Study, we allowed women to refer more than three friends to the study. Heckathorn^1^ recommends no more than three coupons for each recruit in order to maximize the number of possible recruitment waves. A greater number of waves are preferable because they will result in a sample composition that is independent of the initial seed respondents.^1^ Although we only incentivized three referrals, the act of referring more than three could have reduced the number of waves of recruitment for some women and thus resulted in a final sample containing some referral chains that were of insufficient length to be independent of the seed participant. Yet, the practical fact of the matter is that women actively sought additional coupons when their friends did not follow through with their coupon referral, and we believed that pursuit of referrals via our existing networks was a more effective means of achieving our target sample than to continue to recruit additional new seeds. If we had not distributed more than three coupons to those participants, their referral chain would have ended earlier. The use of more than three coupons has been discouraged by researchers conducting HIV surveillance studies and those targeting intravenous drug users because they were concerned that the distribution of too many coupons to each participant would result in the oversaturation of certain networks too quickly, thus reducing the number of waves achieved. However, we believe that this was less of a concern for this study given the nature of the target sample, which we knew to be less densely networked than those described in the HIV RDS populations. In our final WORLDS Study sample, only 3% of the total sample referred more than three women to the study, despite our distribution of more than three coupons to women. Furthermore, each study attained equilibrium, suggesting that the distribution of more than the recommended number of coupons did not undermine our ability to recruit a sample comprising respondents independent of the seed participants.

Although we reached equilibrium in our studies, we had greater numbers of unsuccessful seeds than reported in the literature (94 in Project COPE, 28 in WORLDS). Malekinejad et al.^4^ reported in their review of HIV surveillance studies that, on average, RDS studies had 1.6 (range = 0–19, median = 0) unsuccessful seeds per study, and 59% of studies with available data (n = 86) reported having no unsuccessful seeds. Based on the lack of theoretical rationale for purposefully selecting seed participants who had dense social networks,^1^ we recruited our seeds using newspaper advertisements and word-of-mouth. We also did not stratify our seeds to reflect varying racial and ethnic or age characteristics. It is possible that using targeted strategies for seed selection in future studies would reduce the number of unsuccessful seeds to attain equilibrium more expediently. Ultimately, however, our final analysis indicated that we had sufficiently long chains of referrals to achieve equilibrium in each of the studies, despite some procedures that were not entirely aligned with recommended RDS protocols.

It is possible that the inclusion of a separate comparison group of exclusively heterosexual women in the WORLDS Study may have impacted procedural elements of the study to result in the nonrandom recruitment of peers (see 40 for elaboration on nonrandom recruitment). We began the study with equal numbers of seed participants in each sexual identity category. However, given the much greater numbers of heterosexual women residing in our local region, they were the first group to reach their target enrollment numbers. These heterosexual women were unlikely to refer lesbian and bisexual women to the study based on their referral patterns (i.e. homophily) and their reports of low numbers of lesbian and bisexual women in their social networks. Consequently, we quickly attained our quota of heterosexual women. We subsequently directed lesbian and bisexual women to only refer other lesbian and bisexual friends to the study because our quota of heterosexual women had been reached. The homophily results from the completed study suggested that lesbian and bisexual women had a low propensity to refer heterosexual women; however, this result is distorted due to these procedural aspects of the study that prevented those women from referring their heterosexual female friends to the study. We considered allowing sexual minority women to provide unfettered referrals of heterosexual women to the study, compensating them for the referral but excluding the heterosexual women from the study; however, we rejected that strategy for fear that referrals of sexual minority women would wither in lieu of the greater number of heterosexual friends in their networks.

Meyer and Wilson^14^ argue that RDS may not be a viable sampling method for targeting sexual minorities because individuals in those communities are not as densely networked as other hidden populations; thus, the population parameters in studies of sexual minorities may inaccurately reflect the local population. There is some evidence to suggest that this may have been a weakness of our studies. Participants in each study were asked to provide an estimate of their social network size. Those data were the foundation for the RDSAT analysis to estimate the adjusted population proportions. RDSAT provides a means to calculate the larger local population parameters based on participants’ social network size responses. In the WORLDS Study, for example, the population proportions can be interpreted to suggest that we sampled 32% of the local lesbian population in our study. However, this would extrapolate to mean that a mere 275 women comprise the entire local population of lesbian women. This seems unreasonably low, given the regional census numbers and what we know about the prevalence of sexual minorities in the United States. A similar pattern of findings emerged from the Project COPE data, with resulting population parameters indicating unrealistically low sexual minority populations in our region. One possible explanation for the distorted population parameters is that participants were not able to accurately assess their network size and grossly underestimated the number of sexual minorities in their social networks. Another possibility is that estimating the number of friends based on sexual identity is more difficult to do than participants who are asked in other studies, for example, to estimate the number of IV drug users or jazz musicians in their networks. In other words, the nature of the target attribute may impact participants’ ability to accurately estimate their social network size. Finally, it is also possible that these parameters accurately reflect the networks of the participants in our study and that their networks tend to be clustered but not well connected in the community—another potential weakness of RDS noted by others.^33,34^ Cornwell and Schneider^35^ also found that recruiting participants with large personal networks may be less critical in terms of the effectiveness of RDS than recruiting those who are affiliated with multiple community venues. Smaller cities, unfortunately, do not typically support strong LGBT community venues and thus also may be a factor that limits RDS utility in those settings compared to larger urban areas with more community supports. In sum, the sexual minority population in our small urban region may consist of many smaller clusters that do not overlap in significant ways—a weakness that would support Meyer and Wilson’s warnings about the effectiveness of using RDS to recruit sexual minority populations.

## Suggestions for future RDS studies

Using well-planned implementation strategies, researchers can design their studies to execute RDS effectively to reach sexual minority and other hidden populations. However, as was our goal in this study, transparency and full reporting of RDS procedures is necessary to strengthen the field. RDS serves a critical purpose of recruiting hard-to-reach minority populations, but it is an imperfect science. A growing inventory of potential RDS limitations is emerging in the literature and should be considered by researchers during the early formative stages of their studies. Gile et al.,^32^ for example, suggest a variety of diagnostic strategies that can be used during the planning and data collection phases of studies to improve the quality of the sample and the inferences that can be made based on the final sample. For example, they provide specific questions to ask participants about their RDS experience, allowing for the evaluation of critical aspects of the RDS process (e.g. reciprocity, recruitment bias, finite population effects) using plots and rate calculations. These strategies are designed to allow researchers to dynamically adjust their practices in order to reduce potential biases in the RDS process. Others question standard protocols for error measurement,^21^ variance estimators,^36^ and estimations of bias^37^ to suggest innovative alternative strategies for improving RDS inferences. In conclusion, RDS is imperfect but represents one of the few promising strategies for recruiting hidden populations. We hope that by sharing our RDS experiences and encouraging future researchers to consider alternative approaches, we can contribute to the refinement of a critical tool in the arsenal necessary to combat health disparities among hidden populations, including sexual minorities.
